# Chronic Critical Illness and the Persistent Inflammation, Immunosuppression, and Catabolism Syndrome

**DOI:** 10.3389/fimmu.2018.01511

**Published:** 2018-07-02

**Authors:** Russell B. Hawkins, Steven L. Raymond, Julie A. Stortz, Hiroyuki Horiguchi, Scott C. Brakenridge, Anna Gardner, Philip A. Efron, Azra Bihorac, Mark Segal, Frederick A. Moore, Lyle L. Moldawer

**Affiliations:** ^1^Sepsis and Critical Illness Research Center, Department of Surgery, University of Florida College of Medicine, Gainesville, FL, United States; ^2^Department of Aging and Geriatric Research, Institute on Aging, University of Florida College of Medicine, Gainesville, FL, United States; ^3^Division of Nephrology, Department of Medicine, University of Florida College of Medicine, Gainesville, FL, United States

**Keywords:** sepsis, inflammation, critical illness, PICS, immunosuppression

## Abstract

Dysregulated host immune responses to infection often occur, leading to sepsis, multiple organ failure, and death. Some patients rapidly recover from sepsis, but many develop chronic critical illness (CCI), a debilitating condition that impacts functional outcomes and long-term survival. The “*Persistent Inflammation, Immunosuppression, and Catabolism Syndrome*” (PICS) has been postulated as the underlying pathophysiology of CCI. We propose that PICS is initiated by an early genomic and cytokine storm in response to microbial invasion during the early phase of sepsis. However, once source control, antimicrobial coverage, and supportive therapies have been initiated, we propose that the persistent inflammation in patients developing CCI is a result of ongoing endogenous alarmin release from damaged organs and loss of muscle mass. This ongoing alarmin and danger-associated molecular pattern signaling causes chronic inflammation and a shift in bone marrow stem cell production toward myeloid cells, contributing to chronic anemia and lymphopenia. We propose that therapeutic interventions must target the chronic organ injury and lean tissue wasting that contribute to the release of endogenous alarmins and the expansion and deposition of myeloid progenitors that are responsible for the propagation and persistence of CCI.

## Introduction

Normal protective host responses to infection often become excessive, resulting in the systemic inflammatory response syndrome (SIRS) that can cause a clinical trajectory of refractory shock, fulminant multiple organ failure (MOF), and early in-hospital death. Until recently, this was a common clinical scenario (occurring in >35% of those with sepsis), and a tremendous effort has been directed at understanding and treating this response. Unfortunately, despite dramatic increases in our understanding of sepsis, more than 150 clinical trials testing biological response modifiers directed at SIRS have failed to improve sepsis-associated mortality (1–3). This undoubtedly occurred because the complexity of the human response to sepsis was underestimated, and previous preclinical (bacterial administration and high-mortality peritonitis) (4–9) and early clinical [endotoxin lipopolysaccharide (LPS) administration] (10–13) models did not accurately recapitulate human pathobiology (14–16).

**Figure 1:**
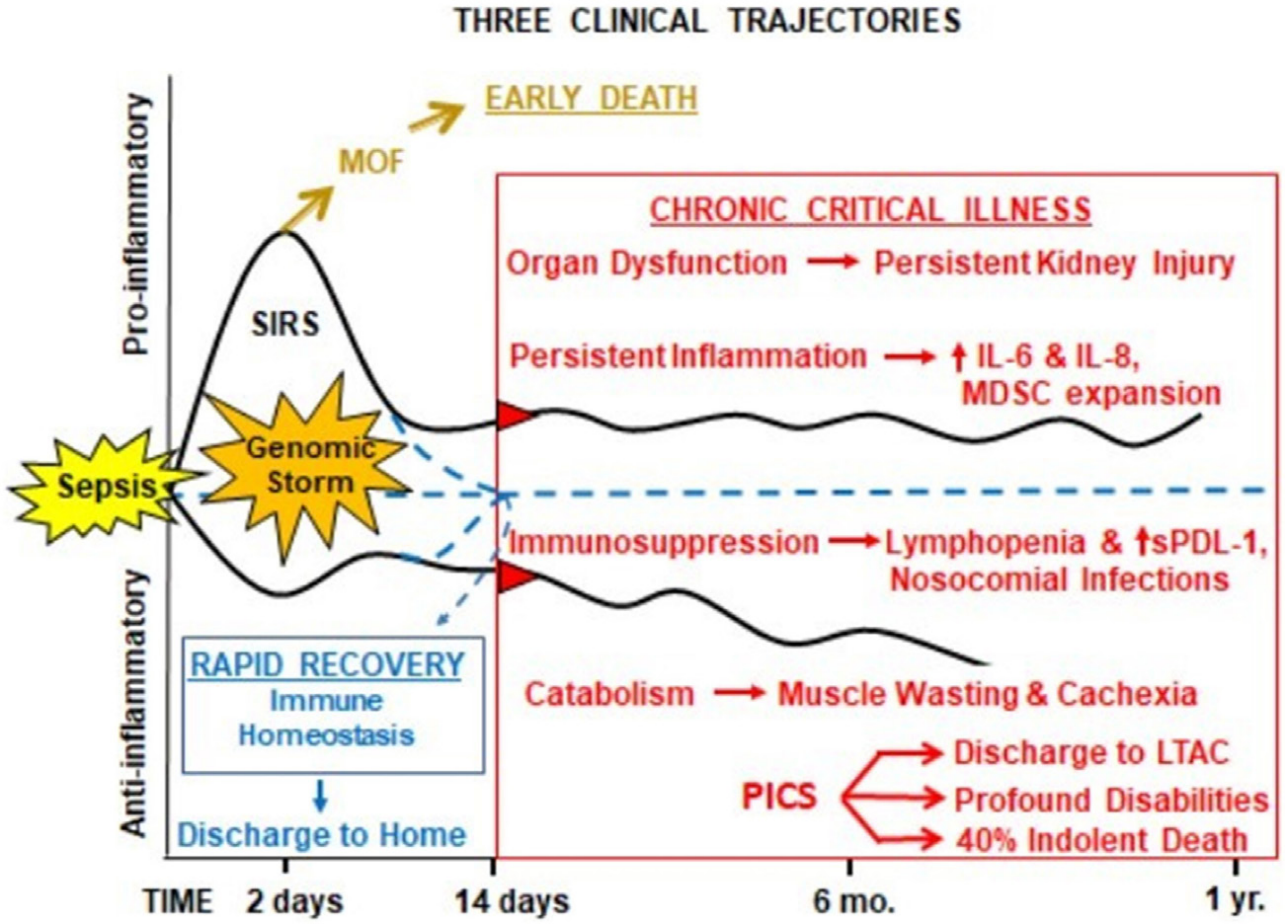
Proposed hypothesis for the PICS. Abbreviations: SIRS, systemic inflammatory response syndrome; MDSC, myeloid-derived suppressor cell; sPDL-1, soluble programmed death ligand-1; LTAC, long-term acute care facility; PICS, persistent inflammation, immunosuppression, and catabolism syndrome.

Despite these efforts, as the result of unprecedented quality improvement to identify sepsis early and provide rapid evidence-based care (17–19), early in-hospital deaths after sepsis have decreased substantially over the last decade. The acute phase of sepsis has been characterized by both a “*genomic storm*” and “*cytokine storm*,” the activation of a plethora of genes that encode inflammatory cytokines, signal transducers, and cell adhesion molecules (20), and subsequent spike of inflammatory cytokines (21). In modern ICUs (Figure 1), severely septic patients are resuscitated through their “genomic storm” characterized by SIRS with organ dysfunction, but now relatively few (<10%) progress into the “MOF/early death” trajectory (18, 22). Our recent studies have shown that as SIRS resolves, roughly half of the sepsis survivors exhibit “rapid recovery” (RAP) from their organ dysfunction and achieve “immune homeostasis.” Unfortunately, the remainder develop “chronic critical illness” (CCI), characterized by prolonged ICU stays (>14 days) and low-grade organ dysfunction (especially kidney injury). CCI encompasses multiple phenotypes, including chronic long-term inflammation as well as immunosuppression and catabolism. Chronically critically ill patients may exhibit either a predominantly pro-inflammatory phenotype or the immunosuppressed phenotype or a combination of the two. CCI patients are often observed to have (a) persistent inflammation [elevated C-reactive protein and increased inflammatory cytokines interleukin (IL)-6 and IL-8] with myeloid-derived suppressor cell (MDSC) expansion, (b) immunosuppression (increased secondary and nosocomial infections and reactivation of latent viral reactivation), and (c) protein catabolism with muscle wasting and cachexia similar to cancer and other chronic inflammatory diseases. MDSCs are a heterogeneous population of immature myeloid cells that accumulate during pathologic conditions such as cancer or sepsis (23). In sepsis survivors, MDSCs are persistently increased, functionally immunosuppressive, and are associated with adverse clinical outcomes (24). CCI patients are commonly discharged to long-term acute care facilities (LTACs) for expensive care because there are no effective interventions (25) and their profound disabilities preclude home care. Here, they experience accelerated aging, induced frailty, sepsis recidivism (requiring re-hospitalization), physical and cognitive disabilities (resulting in dismal life quality), and a high rate of ongoing indolent death (~40% at 1 year) (26, 27). The personal and economic burdens to these patients and their families, as well as the immense costs of this exploding population to society, are immense (1, 28).

## Persistent Inflammation, Immunosuppression, and Catabolism Syndrome (PICS)

**Figure 2:**
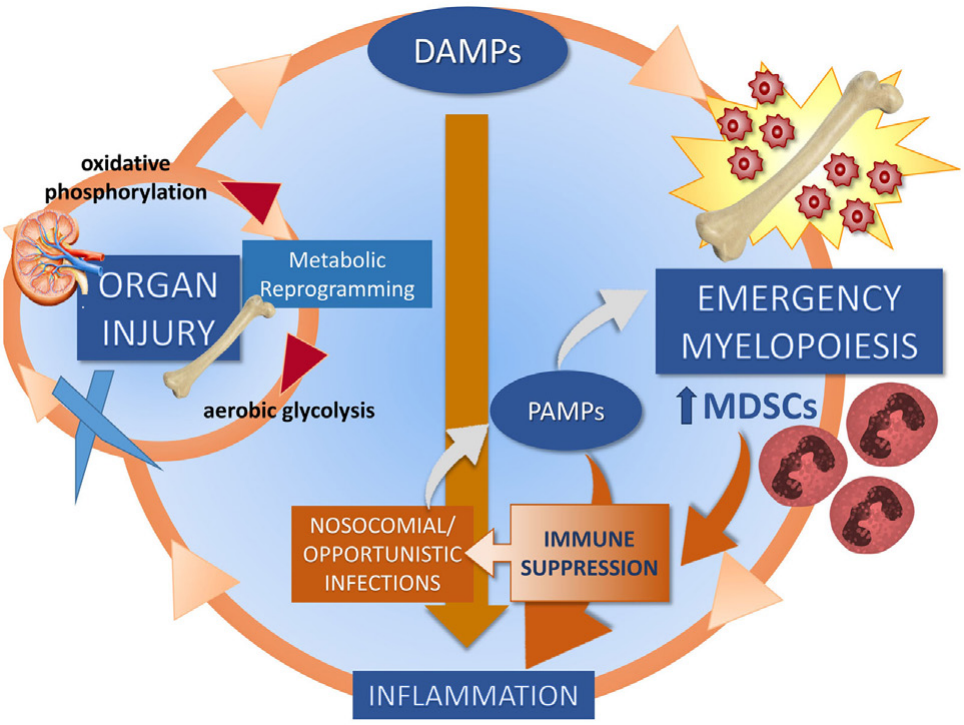
Proposed self-perpetuating cycle of persistent inflammation driving organ injury through a failure of metabolic adaptation, leading to release of endogenous alarmins and bone marrow changes. Three cycles drive CCI: muscle wasting (discussed in Section “Skeletal Muscle as a Target for Oxidant Injury and DAMP Release”), organ injury (discussed in Section “Role of AKI in CCI”), and emergency myelopoiesis (discussed in Section “Abnormal Myelopoiesis and MDSCs”).

In 2012, our group proposed the hypothesis that the underlying pathobiology of CCI in sepsis survivors was a “*Persistent Inflammation, Immunosuppression, and Catabolism Syndrome*” (29). Since 2012, the PICS hypothesis has been validated (30, 31). We now propose, based on new information, that the underlying pathobiology which drives PICS and CCI is a maladaptive self-perpetuating cycle of persistent inflammation involving reduced host protective immunity, continued organ injury and its sequelae, loss of muscle mass and function, changes in bone marrow (BM) function predominated by “emergency myelopoiesis,” and failure of metabolic adaptation (Figure 2) (22, 24, 27, 32, 33). Organ injury results in the release of alarmins, which perpetuates expansion of immunosuppressive myeloid cells, which play a role in ongoing inflammation and muscle wasting which all contribute to the progression of CCI. Recent studies by Bihorac and Segal from our center have shown that new or ongoing acute kidney injury (AKI) is a significant independent predictor of adverse outcomes in sepsis survivors. Not only is the kidney the most commonly injured organ in sepsis (34) but it is by far the most problematic for long-term recovery (29, 35, 36). AKI increases the likelihood of chronic kidney disease (CKD), which is both catabolic and inflammatory. These patients also experience profound muscle wasting with loss of up to 30% of lean body mass within weeks (37, 38).

**Figure 3:**
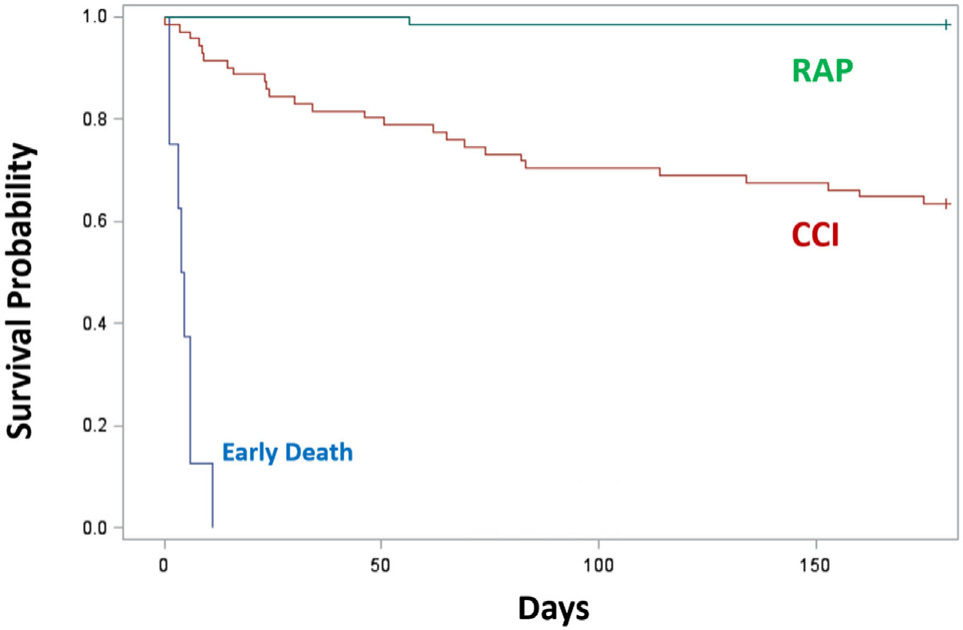
Survival of CCI (*n* = 71) or RAP (*n* = 66) patients 6 months after sepsis. Trajectories were classified as early death (blue), RAP (green), and CCI (red). Kaplan–Meier analysis demonstrated significant differences (*p* < 0.01) in survival between groups. Abbreviations: CCI, chronic critical illness; RAP, rapid recovery.

**Figure 4:**
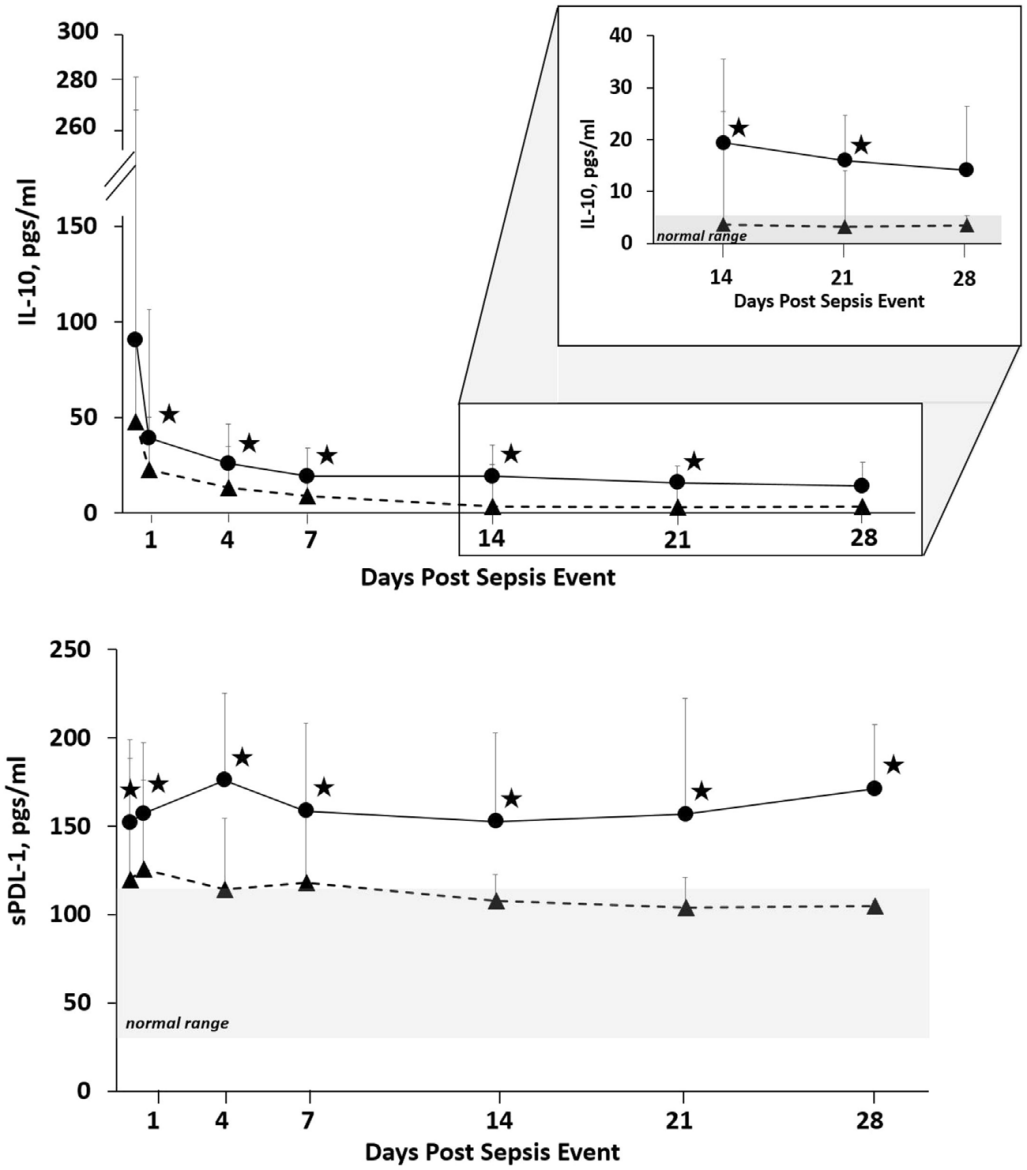
Biomarker concentrations in patients with CCI and RAP. Blood samples were collected at 0.5, 1, 4, 7, 14, 21, and 28 days after sepsis onset. Differences in concentrations between CCI (filled circles) and RAP (filled triangles) cohorts at individual time points are identified with an asterisk at a *p* value less than 0.05 using nonparametric tests. Patients with CCI had evidence of prolonged immunosuppression. Abbreviations: CCI, chronic critical illness; RAP rapid recovery; IL-10, interleukin-10; sPDL-1, soluble programmed death ligand-1.

Since proposing PICS in 2012, several observations have been notable. In regards to the three clinical trajectories (Figure 3), only 6% of septic patients died early (<14 days), 46% experienced rapid recovery (with 6-month survival of 98%), while a notably high 49% progressed into CCI (with 6-month survival of only 63%) (27). Sepsis survivors who developed CCI were significantly older and had more hospital-acquired (rather than community-acquired) infections (27). Importantly, these subjects exhibited not only persistent inflammation, as demonstrated by elevated plasma cytokine concentrations, but also increased immunosuppressive proteins (sPD-L1 or IL-10) (Figure 4) (27). Most (~80%) were discharged to LTACs, developed recurrent infections, and had significant ongoing functional disabilities at 6 months. CCI patients reported worse quality of life and had significantly worse functional status at 6-month follow-up compared with patients who experienced rapid recovery. These results were based on detailed long-term follow-up of CCI patients using the EuroQol-5D weighted utility index (0 = Death, 1 = Full health), the Short Physical Performance Battery (0 = worst performance, 12 = best performance), and the Eastern Cooperative Oncology Group/World Health Organization/Zubrod Scale (0 = Asymptomatic, 5 = Death) (Gardner and Brakenridge, in review).

## The Challenge of CCI

Traditionally, the lean tissue wasting and cachexia frequently observed in CCI has been viewed as a macro-endocrine and cytokine-driven injury stress response (39), and recent studies in CCI survivors have shown biopsy-proven defective mitochondrial biogenesis and myocyte necrosis associated with leukocyte infiltration (38). Inflammation, therefore, is likely causing direct mitochondrial and skeletal muscle myocyte injury, which in turn releases breakdown products that amplify ongoing inflammation. A number of known mitochondrial DAMPs (mitoDAMPs) from skeletal muscle include mitochondrial DNA (mtDNA), HMGB1, and transcription factor A, mitochondrial (TFAM) (40–42). We envision this as a complex, futile, self-perpetuating cycle driven by an inability of organs and tissues to adapt to persistent inflammation, fueled by a continuous release of endogenous alarmins and expansion of MDSCs that infiltrate not only secondary lymphoid organs but also tissues of the reticuloendothelial system. These complex interactions have not been studied systematically.

The current CCI epidemic represents a new and seemingly insurmountable scientific challenge. Sepsis-induced CCI and its bleak long-term outcomes occur in a diverse patient population whose characterization requires both in-hospital and long-term post-hospital discharge follow-up. While these detailed assessments are essential in elucidating the debilitating outcomes associated with CCI, CCI’s natural history is confounded by multiple co-morbid diseases, making a definitive, clinical, mechanistic investigation difficult.

This has made the study of sepsis-associated CCI in animal models challenging. Appropriate, validated laboratory models are required to develop a mechanistic framework describing the variable effects on outcomes in different organs and subgroups of patients (14, 43). Historically, most animal models of sepsis have focused on the early host immune responses in the first few days. This reflected the challenge of treating human sepsis during that acute phase (5, 44). To better study the underlying mechanisms that drive CCI’s progression, continual calibration/validation and improvement of more chronic animal models through ongoing bidirectional bench-to-bedside research will be required. Several groups including ourselves have used a semi-lethal cecal ligation and puncture (CLP) model because it recapitulates the persistent inflammation, weight loss, and immune suppression seen in sepsis survivors (45–50). However, the mechanism(s) driving the process in mice are likely different from those seen in CCI and PICS, because survivors of CLP have a necrotic cecum in addition to an indeterminate nidus of infection. High-dose antibiotics have been attempted to sterilize the peritoneal cavity, but accomplishment of sterility has rarely been demonstrated (51). With that said, some investigators have looked at the long-term outcomes in murine survivors of CLP sepsis, and demonstrating myeloid cell expansion, inflammation, and immune suppression (52–54).

In addition, most investigators have used juvenile or young adult in-bred mice; however, sepsis is a disease of the elderly. Efron et al. have used aged mice and clearly shown that not only do they differ from young mice at baseline but they also respond differently to sepsis (14). We are also beginning to understand the effects of comorbidities on the sepsis response. Delano has shown that preexisting diabetes exaggerates the host response to sepsis (55). This type of information will be crucial in the future development of multimodal interventional trials.

## Alarmins and Danger-Associated Molecular Patterns (DAMPs) Drive the Persistent Inflammation

Alarmins have been identified as important mediators of persistent inflammation in CCI. There are two sources of alarmins and both are recognized by the same pattern-recognition receptors on immune and parenchymal cells that perpetuate ongoing inflammation. The first source is exogenous pathogen-associated molecular patterns elaborated during nosocomial infections and reactivation of latent viral infections (43). Our recent studies show that 61% of sepsis survivors suffering from CCI experience one or more secondary nosocomial infections compared with 18% in RAP survivors (33). Hotchkiss reported that 100% of patients spending 14 days in the ICU show evidence of viral reactivation (56). The second source of alarmins is the constant release of endogenous DAMPs from injured organs and inflammatory cells (57). These danger signals represent both nucleic acids and cellular proteins that are released upon cell death, as well as proteins or nucleic acids that are actively secreted in response to cellular stress (57, 58). Multiple studies have demonstrated that several of these DAMPs, including nuclear (nuc) DNA, HMGB1, and S100 are significantly elevated in sepsis survivors, especially during their entire hospitalization (59–62). We hypothesize that the two primary sources of these endogenous DAMPs are the kidney (with AKI progressing to CKD) and wasting of skeletal muscle, the largest and most labile protein reserve in the body.

## Abnormal Myelopoiesis and MDSCs

Sepsis initiates an emergency myelopoiesis response (24, 63). Cytokine and chemokine release (64–66), along with adrenergic stimulation (67, 68), promotes the rapid release of myeloid populations from BM and secondary lymphoid organs. This release of both mature and immature neutrophils (PMNs) is an essential requirement for the initial control of an invading pathogen, but it can also cause collateral organ injury. This release also creates a void in the BM niche that stimulates expansion of hematopoietic stem cells and other early progenitors (69). Myelopoiesis is favored at the expense of both lymphopoiesis and hematopoiesis, which explains in part the persistent lymphopenia and chronic anemia seen in sepsis survivors (66, 70).

In cases of CCI in which alarmin release continues unabated, however, emergency myelopoiesis persists and myeloid cells are released with an immature phenotype (71, 72). We have seen in a chronic murine CLP model that by 7 days post-sepsis, up to 95% of BM cells are myeloid cells, mostly immature and functional like MDSCs (53). Interestingly, McCall has shown that the phenotype of these cells evolves over time, and the myeloid cells become more immunosuppressive with time (73). These cells not only overwhelm the BM but also significantly infiltrate the spleen, lymph nodes, reticuloendothelial tissues (such as lung and liver), and likely also skeletal muscle and brain (53, 74). MDSCs are generally sorted into two phenotypes based on cell surface markers: granulocytic MDSCs are CD11b^+^Ly6G^+^Ly6C^low^ and monocytic MDSCs are CD11b^+^Ly6G^−^Ly6C^high^ (75). MDSCs have been implicated in immunosuppression in sepsis *via* IL-10 production and inhibition of T-cell response and proliferation (76). Not only are MDSCs potently immunosuppressive toward macrophages and CD4^+^ and CD8^+^ T-cells, they are also pro-inflammatory, producing oxidation, and peroxidation products, as well as nitric oxide, all of which are potentially damaging to parenchymal cells and promote inflammation (66, 76). We have recently demonstrated that in sepsis survivors, there is rapid and sustained appearance of MDSCs in the blood (24). More importantly, we have shown that CCI sepsis survivors have persistently elevated MDSCs that are strong independent predictors of nosocomial infection and hospital discharge to LTACs (24, 27, 33). Others have confirmed our results and demonstrated that sepsis survivors have elevated levels of MDSCs and similarly have a high incidence of secondary nosocomial infections (77, 78).

## Role of AKI in CCI

The kidney is likely the most critical organ related to long-term recovery from sepsis. Our previous work found a strong association between sepsis and AKI. Not only are patients with AKI more likely to develop sepsis (34, 79–81), but AKI progressing to CKD is a major factor that perpetuates organ dysfunction in sepsis, leading to CCI and decreased survival (29, 35, 36). Renal tubule epithelial cells are highly susceptible to intrinsic oxidative stress. During sepsis, necrotic tubule epithelial cells and PMNs release DAMPs that activate PPR toll-like receptors (TLRs). Others have shown that patients with sepsis and AKI have increased levels of urinary DAMPs (82). In addition, urinary cellular RNA from sepsis patients reveals overexpression of several DAMP receptors. The local and systemic influx of DAMPs leads to secretion of chemokines by renal parenchymal cells and dendritic cells (DCs) which promotes a further local PMN-dependent inflammatory response (83–85) as well as distant systemic effects on other organs (36, 86–90).

In addition to being constitutively expressed in renal tubule epithelial cells, TLRs are further upregulated in AKI *via* epigenetic remodeling which leads to an exaggerated cytokine production in response to LPS and lipoteichoic acid, causing a renal “hyper-responsive” state (91). The cytokine efflux into the systemic circulation, together with the decrease in renal clearance, leads to exaggerated systemic inflammation and systemic organ injury. In trauma patients with AKI, we observed an increase in plasma levels of IL-8, chemokine (C-X-C motif) ligand 1 (CXCL1), monocyte chemoattractant protein-1, and macrophage inflammatory protein 1-beta (92). In both animal models and human studies, distant lung injury after AKI was associated with an increase in plasma levels of CXCL1, IL-6, and IL-8 within 2 h after AKI onset (93, 94). Our data demonstrate persistent elevation of both pro-inflammatory and immunosuppressive cytokines among patients with persistent AKI.

There is currently a dearth of knowledge explaining why the kidney is so susceptible to inflammatory injury and why CCI patients fail to recover from renal dysfunction. Large numbers of immune cells (such as DCs, macrophages, and few lymphocytes) reside in the kidney (95). We propose that during sepsis and CCI, the kidney is infiltrated by MDSCs that bring with them oxidative and immunosuppressive properties (24, 30, 76). Each nephron selectively filters small molecules such as DAMPs and pathogenic antigens and together the kidneys filter approximately 180 l of fluid per day, filtering the entire blood volume over 30 times daily (96). Thus, renal DCs and renal lymph nodes are exposed to DAMPS, pathogens, and antigens in the blood over 30 times more frequently than any other tissue. The kidney hosts many different cell types expressing a subset of TLRs (1–6) and can thus respond to DAMPs to induce innate immune responses (97).

## Skeletal Muscle as a Target for Oxidant Injury and DAMP Release

The skeletal muscle system is the largest, most labile protein reserve in the body. Sepsis induces catabolism characterized by profound muscle wasting, reflecting breakdown of myofibrillar proteins, decreased protein synthesis, increased mitochondrial dysfunction, and the release of potential pro-inflammatory degradation products from the large numbers of myocyte mitochondria (40, 98, 99). In long-term follow-up studies, muscle atrophy has been shown to cause severe functional disabilities in CCI survivors (100, 101). Currently, it is unknown what the precise role is of mitochondrial dysfunction and inflammation in sepsis on skeletal muscle wasting and long-term outcomes.

A novel emerging role of skeletal muscle is its ability to regulate inflammation not only locally but also systemically. Increased catabolism of skeletal muscle during sepsis, either through oxidant injury-induced cellular apoptosis or autophagy, can stimulate an immune response *via* cellular constituents being released into the circulation and acting as DAMPs. Infiltration of skeletal muscle with myeloid cell populations, including potentially MDSCs, is recognized in skeletal muscle injury and sepsis-associated skeletal muscle wasting (38, 102–105). Fragments such as mtDNA, ATP, TFAM, *N*-formyl peptides, HMGB1, succinate, and cardiolipin are known mitoDAMPs and can function as endogenous alarmins to propagate chronic inflammation (40). These factors, which can act systemically, may be released during skeletal muscle injury or critical illness-associated muscle wasting. TFAM, one of the potential alarmins released during skeletal muscle damage, has divergent local and systemic functions (106). Acting locally within tissues, TFAM binding to the D-loop in mtDNA is essential for upregulation of mtDNA replication, resulting in increased mtDNA copy number. In striking contrast, if mtDNA or TFAM is released into the cytosol or circulation, it can activate TLR9 pathway factors (41, 107). Animal models of sepsis-induced cardiac inflammation show that at least partially mtDNA–TLR9–RAGE pathway is involved and activated but can be inhibited by a TLR9 inhibitor (41).

## Conclusion

Sepsis induces a genomic and cytokine storm that can cause variable host responses in the long term. A substantial portion of sepsis survivors go on to develop CCI, a debilitating condition with profound personal and social costs. CCI is multifactorial and complex, and better understanding is necessary to improve long-term outcomes from sepsis. We hypothesize that CCI is initiated by an early genomic storm, organ injury, and skeletal muscle wasting that leads to a DAMP-driven pro-inflammatory expansion of immature myeloid cells, and infiltration of MDSCs in organs and tissues. The continual release of DAMPs from CKD or muscle wasting propagates the PICS response in sepsis survivors. We propose further work in developing appropriate animal models to study CCI, AKI, and CKD, and targeted interventions to alter the disrupted homeostasis of metabolic reprogramming after sepsis.

## Author Contributions

RH, SR, JS, HH, AG, and LL drafted the manuscript. SB, PE, AB, MS, FM, and LM provided critical revisions. All the authors made substantial contributions to the conception and design of the work, approved the submitted version of the manuscript, and agreed to be accountable for all aspects of the work.

## Conflict of Interest Statement

The authors declare that the research was conducted in the absence of any commercial or financial relationships that could be construed as a potential conflict of interest.
